# Foot-and-mouth disease virus O/ME-SA/Ind 2001 lineage outbreak in vaccinated Holstein Friesian cattle in Saudi Arabia in 2016

**DOI:** 10.1080/01652176.2018.1539568

**Published:** 2019-01-09

**Authors:** Maged Gomaa Hemida, Walid Rizk EL-Ghareeb, Fahad Al-Hizab, Abdelazim Ibrahim

**Affiliations:** a Department of Microbiology and Parasitology, College of Veterinary Medicine, King Faisal University, Al-Hasa, Saudi Arabia;; b Department of Virology, Faculty of Veterinary Medicine, Kafrelsheikh University, Kafelsheikh, Egypt;; c Department of Veterinary Public Health, College of Veterinary Medicine, King Faisal University, Al-Hasa, Saudi Arabia;; d Food Control Department, Faculty of Veterinary Medicine, Zagazig University, Al Sharqia Governorate, Egypt;; e Department of Pathology, College of Veterinary medicine, King Faisal University, Saudi Arabia;; f Department of Pathology, College of Veterinary Medicine, Suez Canal University, Ismailia Governorate, Egypt

**Keywords:** Cattle, foot-and-mouth disease, PCR, pathology, serotype O, Saudi Arabia

## Abstract

**Background:** Foot-and-mouth disease virus (FMDV) is a highly contagious viral infection of large ruminants. Despite the massive application of vaccines against FMDV, several outbreaks are still being reported in Africa and Asia.

**Aim:** To perform molecular characterization of FMDV in an outbreak among a cattle herd Saudi Arabia in 2016. This herd had been vaccinated with a polyvalent FMDV vaccine.

**Methods:** To investigate this outbreak, we collected specimens from 77 animals showing typical clinical signs of FMDV. Specimens including sera, nasal swabs, and tissues (tongue, coronary bands, hooves, and hearts) were collected. We tested the collected cattle sera for the presence of FMDV antibodies with commercial ELISA kits. In addition, we tested the swabs for the presence of the most common FMDV strains (O, A, Asia-1 and SAT-2) with RT-PCR using serotype-specific oligonucleotides.

**Results:** Serology showed that 22% of the tested sera were positive. Molecular testing of the examined swabs confirmed that 24% of the tested animals were positive. Our sequencing analysis confirmed that the circulating strains of FMDV belonged to FMDV serotype O. The phylogenetic tree based on the FMDV-VP-1 gene revealed high nucleotide identity between the circulating strains and the Bangladesh strain (99%). These strains were distinct (shared 89% nucleotide identity) from the FMDV-O strains used for the preparation of the vaccine administered to the animals in this herd. Moreover, they had 7% nucleotide difference between the FMDV-O strains reported in Saudi Arabian in 2013.

**Conclusion:** More in-depth molecular characterization of these FMDV strains is warranted.

## 1. Introduction

Foot-and-mouth disease virus (FMDV) is one of the most devastating viral infections of cloven-hooved animals (Ranjan et al. 2018). FMDV infection usually results in high economic losses for the animal industry for many reasons including a sharp drop in milk yield, decrease in the feed conversion rate, lameness of the affected animals and death, particularly in young animals (Ranjan et al. 2018). FMDV belongs to the genus Aphthovirus and the family *Picornavirdae*. The viral genome is a single-strand positive sense RNA. The viral genome ranges from 6.9 to 8.3 kilobases in size and is flanked by two untranslated regions at its 5′ and 3′ ends. There is a polyadenylation tail downstream of the 3′UTR region (Fry et al. 2005). The 5′ end is a large fragment of the viral genome, which is divided into several fragments with various functions during the viral replication (Fry et al. 2005). The virion is icosahedral in symmetry and consists of 60 capsomeres. These capsomeres have four structural proteins in each (VP-1 through VP-4). The first three proteins, VP-1 through VP-3, are expressed on the outer service of the virus, while VP-4 is located inside the virion (Fry et al. 2005). There are seven distinct immunologic strains of FMDV called South African Territories, 1, 2, and 3, in addition to serotypes A, O, and Asia 1 (Carrillo et al. 2005). There is no cross-protection between the different serotypes (Brito et al. 2014; Cao et al. 2014); thus, infection or vaccination with one FMDV strain does not provide protection against other strains (Kitching et al. 1989). Based on the VP-1-based phylogenetic trees, the FMDV A serotype is divided into 10 major genotypes (I-X). Furthermore, there are 10 topotypes of the FMDV O serotype. Moreover, there are six genotypes of the FMDV Asia 1 serotype (I–VI) (Valarcher et al. 2009). Although various FMDV vaccines are available in most countries, ongoing outbreaks are still reported in some of these animal populations (Eble et al. 2015). Also in Saudi Arabia, several FMDV outbreaks have been reported (Samuel et al. 1988; Abd El-Rahim et al. 2016; Mahmoud and Galbat 2017). One study during 1986–1987 reported the detection of serotype A in some animals from Saudi Arabia and Iran (Samuel et al. 1988). The circulating strains were closely related to each other and were classified as FMDV A22 variant (Samuel et al. 1988). Another study analyzed the available data on the vaccination regimes of some dairy farms in Saudi Arabia (Woolhouse et al. 1996). This study developed a mathematical model for predicting the protective efficacy of those FMDV vaccines and their intervals of administration. This study revealed that the neither the vaccines used nor the vaccination interval used provided a high degree of protection for the herds against FMDV field infection (Woolhouse et al. 1996). A new study evaluated the effects of importing large and small ruminants from Africa on the introduction of new FMDV strains to the Kingdom of Saudi Arabia (Abd El-Rahim et al. 2016). They found that almost 50% of the tested sheep and cattle had FMDV antibodies (Abd El-Rahim et al. 2016). A recent serosurveillance performed among some imported foreign breeds of sheep revealed the presence of antibodies against FMDV serotype O and against the Peste-des-petits-ruminants virus (PPRV) (Mahmoud and Galbat 2017). The major goals of the current study were to perform molecular characterization of an FMDV outbreak among a cattle herd in Eastern Saudi Arabia in 2016 as the currently circulating FMDV strains in Saudi Arabia are not well characterized at the molecular level.

## 2. Materials and methods

### 2.1. Outbreak description

We investigated an FMDV outbreak in a cattle herd located in Eastern Saudi Arabia during the winter of 2016. We approached this outbreak by examining 77 cows out of 780 and collected serum and nasal swabs from these animals for further testing.

### 2.2. Ethical animal research assessment

All animal utilization and sample collection was carried out as per The King Abdul-Aziz City of Science and Technology, Royal Decree No. M/59, (http://www.kfsh.med.sa/KFSH_WebSite/usersuploadedfiles%5CNCBE%20Regulations%20ENGLISH.pdf). This animal utilization protocol was amended by the King Faisal’s University Animal Ethics and the National Committee of Bioethics (NCBE).

### 2.3. Herd description

**Figure 1.**
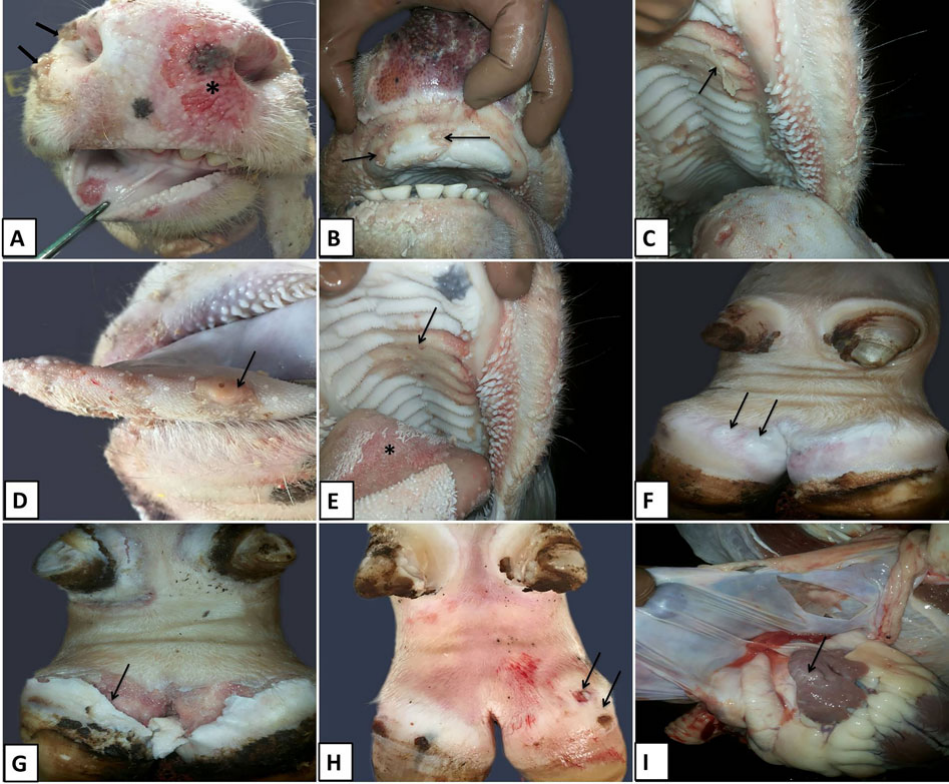
Clinical and pathological pictures of some FMDV infected cattle in Eastern Saudi Arabia in 2016. (A) Note the hyperemic and slightly eroded areas on the muzzle and inner side of the lower lip (asterisk) with two vesicles on the nares (arrows). (B) Ruptured vesicles on the dental pad (arrows). (C) Sloughed hard palate leaving an ulcerated surface (arrow). (D) Single vesicle on the side of the tongue. (E) Large ulcerated areas on the dorsal surface of the tongue (asterisk) and on the hard palate (arrow). (F) Multiple vesicles on the coronary band (arrows). (G) Ruptured coronary band vesicles. (H) Ulcerated areas on the skin above the coronary band (arrows). (I) Focal gray area of myocardial necrosis (arrow).

All animals under study were Holstein Friesian cattle. Both adult cattle and young calves were housed within the same barn with some partitions. The herd consisted of 4000 cows including 1250 calves. Lactating animals were milked four times per day. Animals showing obvious FMDV clinical signs were housed in a quarantine area. We examined the quarantined animals and reported the clinical signs (Figure 1). Samples were collected from 77 animals before slaughtering. During carcass inspection after slaughtering, we selected the organs showing the typical FMDV lesions from each animal. These organs were subjected to further processing for the histopathology technique as described in the M&M section.

### 2.4. Specimens and processing

#### 2.4.1. Sera

We collected 77 whole blood samples from the selected animals in this outbreak. In addition, we tested 92 archived cattle serum samples from our laboratory from 1993. These specimens were collected as part of large nationwide surveillance for Rift Valley fever across Saudi Arabia (Al-Afaleq et al. 2003). We placed the collected blood samples at 4 °C overnight and centrifuged these samples at 5000 RPM for 5 min. We separated the sera with pipettes and then transferred them to sterile tubes. We heated the sera at 56 °C for 30 min to inactivate the nonspecific inhibitors. The collected sera were stored at –20 °C for further testing.

#### 2.4.2. Swabs

We collected 77 nasal swabs from the investigated cattle herd. Swabs were collected on a transport medium containing Dulbecco-modified Eagle’s medium, 10% fetal bovine serum, and an antibiotic cocktail (100 U/mL penicillin and 100 μg/mL streptomycin). We processed the collected swabs by centrifugation at 5000 RPM for 5 min on a cooling centrifuge. The supernatants were separated and stored at –80 °C for further testing.

#### 2.4.3. Tissues

We examined 45 animals at necropsy and selected the animals showing typical FMDV lesions. We collected tissue specimens from affected organs (tongue, lips, dental pad, and the skin of the coronary bands) of the suspected FMDV-infected animals and processed these tissues for histopathological examination. Briefly, these tissue specimens were immediately immersed in 10% neutral buffered formalin. Fixed tissues were processed in increasing gradients of ethyl alcohol and xylene and then embedded in paraffin blocks. Five micrometer paraffin sections were cut and stained with H&E stain for histopathological examination.

#### 2.4.4. Enzyme-linked immunosorbent assay (ELISA)

We tested the collected sera for the presence of FMDV antibodies by using the ID Screen^®^ FMD NSP Competition kits (FMDNSPC-10P) (ID Vet Genetics, Grabels, France). The ELISA technique was carried out according to the kit’s instructions and has been previously described (OIE 2012).

#### 2.4.5. RNA extraction

We extracted the total viral RNA from the collected swabs by using QIA-Amp RNA extraction kits (Qiagen, Hilden, Germany) according to the kit’s instructions. The RNA concentration was measured with the Nanodrop machine (Thermo Scientific NanoDrop 2000, Applied Biosystems, Foster City, CA), and then the RNA samples were stored at –80 °C until testing.

#### 2.4.6. Oligonucleotides

We used partial FMDV VP-1 gene oligonucleotides to test the collected specimens for the common FMDV strains (O, A, Asia 1, and SAT 2) previously reported in in Eastern Saudi Arabia in 2016. We used the previously published specific FMDV oligonucleotides (Le et al. 2011). The details of these primers are listed in Table 2.

**Table 1.** Oligonucleotides used for RT-PCR amplification of the partial FMDV VP-1 gene from Eastern Saudi Arabia in 2016.

No.	Primer name	Sequences 5′ 3′	Size (bp)	References
1	FMDV-VP-common-R^a^	CATGTCYTCYTGCATCTGGTT	NA	Le et al. (2011)
2	FMDV-VP1-O-F	AGATTTGTGAAAGTDACACCA	670	
3	FMDV-VP1-A-F	CTTGCACTCCCTTACACCGCG	427	
4	FMDV-VP1-Asia-1-F	GCGSTHRYYCACACAGGYCCGG	535	
5	FMDV-VP1-SAT-F^b^	CCACATACTACTTTTGTGACCTGGA	718–730	Vosloo et al. (1992)
6	FMDV-VP1-SAT-R^b^	ACAGCGGCCATGCACGACAG		

a Reverse primer used for the amplification of FMDV VP-1 of three serotypes (O, A, and Asia 1).

b Primers used for the amplification of the three FMDV SAT groups (SAT-1, SAT-2, and SAT-3).

**Table 2.** Summary of the molecular and serological surveillance of FMDV in Eastern Saudi Arabia.

Type	Total	(+Ve)	(–Ve)	% (+Ve)
Swabs	77	13	54	24
Sera	77	17	51	22
Archived sera	92	48	44	52

#### 2.4.7. Synthesis of the cDNAs and PCR reactions

The extracted RNA samples were subjected to two-step RT-PCR. The technique was carried out as previously described (Brunner et al. 2014) with some modifications. The RT-PCR reactions were performed in 20 µL reactions including 2 µL of the dsRNA samples, 1 µL of the sense FMDV primer (Brunner et al. 2014), and 1 µL of the Moloney murine leukemia virus reverse transcriptase (M-MLV, TakaRa, Beijing, China). The synthesized complementary DNA (cDNA) was amplified by RT-PCR. Fifty microliter reactions were prepared, containing 1 µL each of the template cDNA, both FMDV sense and antisense primers, and the PCR master mix and 1 µL of Taq DNA polymerase (TakaRa, Beijing, China). We used the following parameters: initial denaturation for 5 min at 95 °C; then 94 °C for 1 min; annealing at 55 °C for 30 s repeated for 30 cycles; and a final extension at 72 °C for 10 min.

#### 2.4.8. Gel electrophoresis

Ten microliter of the amplified RT-PCR reactions were separated by 1% agarose gels containing SYBR^®^ Safe DNA Gel Stain (Invitrogen, Thermo Fisher Scientific, Waltham, MA). Amplified reactions were visualized under ultraviolet light. The gel pictures were photographed with the gel documentation system (Bio-Rad Laboratories, Inc., Hercules, CA).

#### 2.4.9. Purification of the amplified PCR amplicons

The target amplified PCR bands were excised from the gel and purified using the QIA-quick Gel Extraction Kit (Cat No/ID: 28704), according to the kit’s instructions. The purified reactions were eluted in a 50-μL elution buffer.

#### 2.4.10. Sequencing and sequencing analysis

We selected some positive RT-PCR specimens and sequenced them with a Sanger approach. Sequencing of the amplified PCR products was performed using the Applied Biosystems^®^ 3500 sequencing machine. The purified PCR products were sequenced in both directions using the original oligonucleotides used in the PCR amplification. We assembled the obtained sequences into one contig by using the Sequencher 5.4.6 sequencing analysis software (^©^2017 Gene Codes Corporation, Ann Arbor, MI) and performed nucleotide blast in NCBI (https://blast.ncbi.nlm.nih.gov/Blast.cgi?CMD=Web&PAGE_TYPE=BlastHome). These sequences were aligned and compared to other FMDV sequences available in GenBank.

#### 2.4.11. Phylogenetic analysis

We constructed phylogenetic trees (maximum likelihood) based on the obtained sequences. Multiple alignments of these sequences with other sequences from GenBank were performed using the Mega-7 package, and phylogeny was performed using the neighbor-joining method with 1000 bootstrap replicates, as previously described (Kumar et al. 2016).

#### 2.4.12. Statistical analysis

We applied a nonprobability sampling strategy for our sample collection with an incidental assignment approach, as previously described (Smith 1983). Results considered significant when the *P* value is less than 0.05.

## 3. Results

### 3.1. Outbreak description

We observed a recent FMDV outbreak in a dairy herd in Eastern Saudi Arabia. The examined animals showed typical signs of FMDV infection. The affected cattle population showed a high morbidity (85%) with minimal rates of mortality (<1%). The inspected animals had high fever (above 39.5 °C), increased respiratory rates, inappetence, recumbency, and profuse salivation.

### 3.2. Postmortem investigation

Gross and postmortem examinations of the affected animals revealed the presence of lesions in different parts of the body, such as the external nares, muzzle, lips, dental pad, gums, hard palate, tongue, and coronary bands. The lesions first appeared as hyperemic shallow eroded areas, and then became pale and blanched. Vesicle formation was usually noticed in many locations, especially on the dorsum of the tongue, and vesicles ranged from 0.5 to 2 cm in diameter. Vesicles were ruptured leaving an ulcerated surface that was covered with a whitish pseudomembrane, representing the remnant of the vesicle wall. Occasionally, in severe cases, the hooves were sloughed from the digits exposing the underlying surface. Cross-sections of the heart revealed a moderate amount of clear straw yellow fluid in the pericardial sac and the presence of an irregular grayish-white area of necrosis within the myocardium.

### 3.3. Histopathology of tissues from FMDV-infected animals

**Figure 2.**
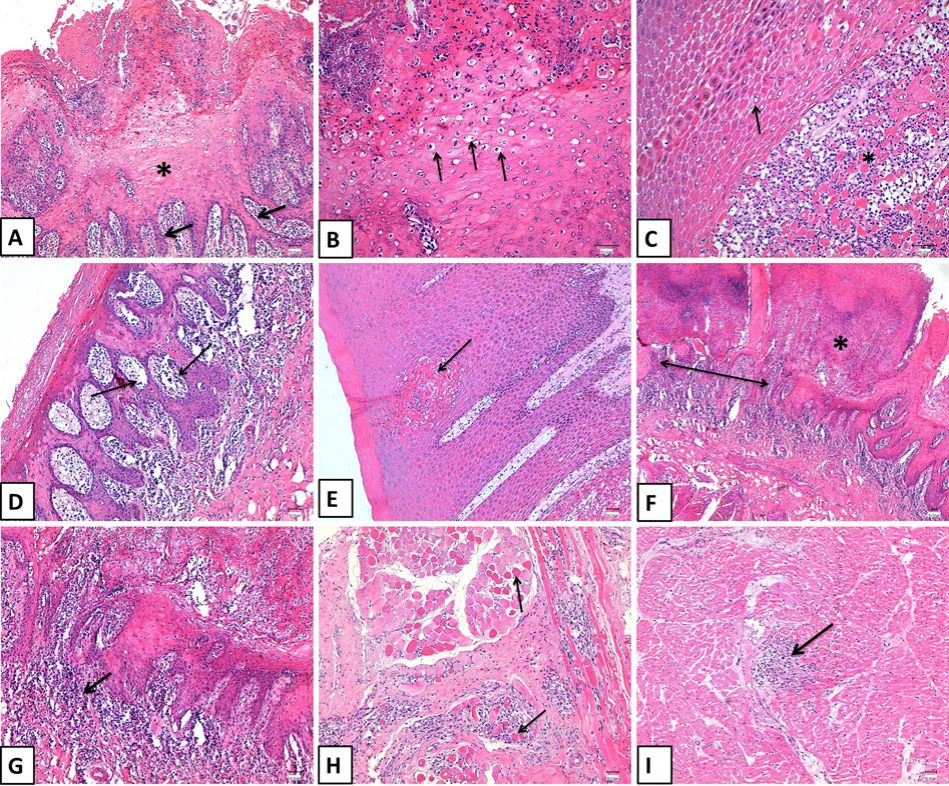
Histopathological pictures of some FMDV-infected cattle in Eastern Saudi Arabia in 2016. H&E-stained sections from animals showing clinical signs of FMDV (Bar =50 µm). (A) The epithelium of the coronary band shows acanthosis (asterisk) with elongation of rete ridges (arrows). (B) A higher magnification of the previous images demonstrates intracellular edema in the cells of stratum spinosum (arrows). (C) Note the presence of prominent intercellular bridges (arrow) with dissociation of keratinocytes (asterisk). (D) Multiple microvesicles are seen within the epidermis of the coronary band (arrows). (E) A focal area of coagulative necrosis (arrow) is located within the epidermis. (F) The lingual epithelium is ulcerated (double-headed arrow) and covered with cellular and karyorrhectic debris (asterisk). (G) The lamina propria of the tongue is moderately infiltrated with inflammatory cells, mostly lymphocytes (arrow). (H) The cardiomyocytes exhibit a hypereosinophilic cytoplasm with a loss of striation. (I) A focal area of the myocardium is replaced with lymphocytes.

Various histopathologic changes were observed in the tissue specimens (tongue, lips, dental pad, and skin of the coronary bands) collected from animals showing typical clinical FMDV infections. These lesions were found separately or collectively in the same specimen. The stratified squamous epithelium was moderately thickened and irregular because of hyperkeratosis and acanthosis, with anastomosing rete ridges (Figure 2(A)). Many cells of stratum spinosum had clear vacuoles within their cytoplasm and hydropic degeneration, indicating intracellular edema (Figure 2(B)). Intercellular edema was also noticed as prominent intercellular bridges and spongiosis (Figure 2(C)). It was severe enough to dissociate keratinocytes from each other through keratinolysis. Microvesicles were seen multifocally within the stratum spinosum as small empty spaces that were sometimes filled with acellular homogenous eosinophilic fluid (Figure 2(D)). Keratinocytes were randomly necrotic, as evidenced by a hypereosinophilic cytoplasm with pyknotic nuclei (Figure 2(E)). The epithelium was eroded and ulcerated in several locations and was overlaid with a serocellular crust composed of cellular and karyorrhectic debris, neutrophils, and fibrin (Figure 2(F)). The dermis/submucosa was slightly edematous and was infiltrated with many inflammatory cells including lymphocytes, macrophages, and neutrophils (Figure 2(G)). Moreover, neutrophils were observed transmigrating across the stratified epithelium or forming aggregations of intracorneal pustules. The skeletal myocytes of the tongue were occasionally infiltrated with few inflammatory cells, and they showed a variable degree of degeneration and necrosis. The myocardium exhibited multifocal areas of cardiomyocyte degeneration and necrosis that was associated with fragmentation, a hypereosinophilic cytoplasm, pyknotic nuclei and a loss of striation (Figure 2(H)). The lost fibers were replaced by lymphocytes and histiocytes (Figure 2(I)).

### 3.4. Molecular surveillance of FMDV

**Figure 3.**
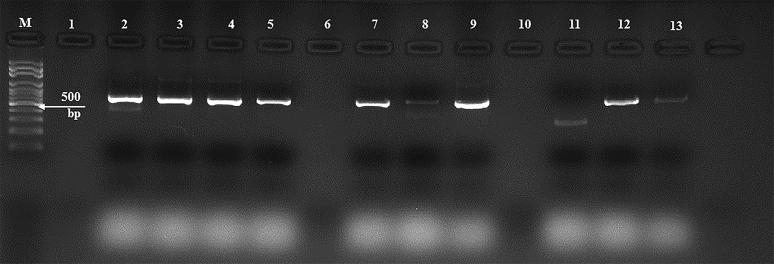
Agarose gel electrophoresis of some RT-PCR-tested nasal swabs from cattle for FMDV in 2016 in Eastern Saudi Arabia. Some RT-PCR results of selected specimens collected from the nasal swabs of FMDV-infected animals. Lane (M) DNA marker, (100 bp); lane (1) empty well; lanes (2–13) purified PCR products of the partial FMDV VP-1 gene. The positive amplicons are 641 bp in length.

We tested the 77 collected nasal swabs for the presence of the FMDV nucleic acids with RT-PCR using the VP-1 oligonucleotides shown in Table 1. We tested these specimens for the most common FMDV serotypes previously reported in Saudi Arabia including O, A, Asia 1, and SAT 2. Figure 3 shows an example of the gel-based PCR testing for some of the tested nasal swabs of cattle. The amplified amplicons were 641 nucleotides in length (Figure 3). Our results clearly showed that 13 of the 77 animals tested were positive (24%) (Table 2).

### 3.5. Molecular characterization of circulating FMDV strains in Eastern Saudi Arabia in 2016

**Figure 4.**
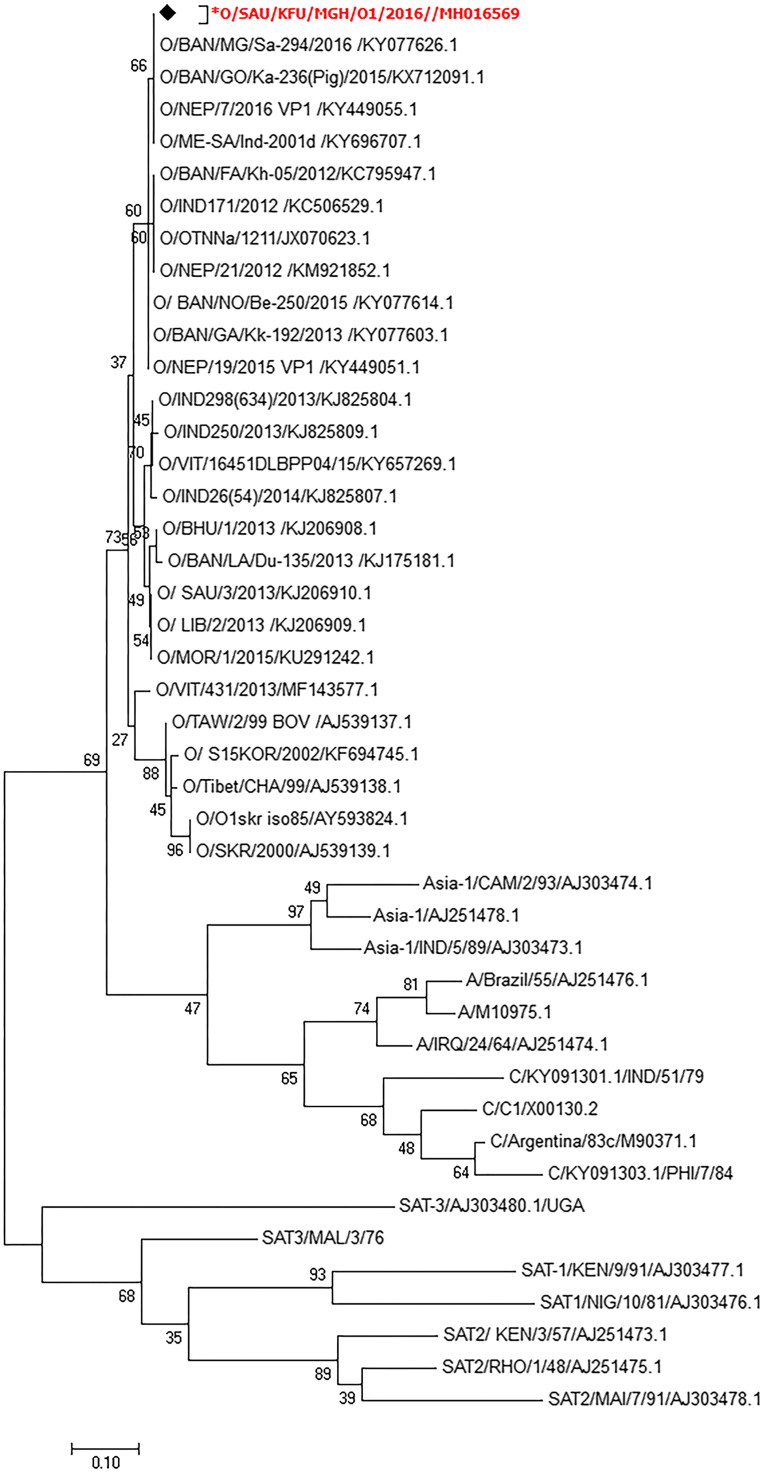
Molecular identification of circulating FMDV strains in a large ruminant herd in Eastern Saudi Arabia in 2016. The maximum likelihood phylogenetic tree of circulating FMDV strains in Eastern Saudi Arabia in 2016 in a dairy herd. This phylogram shows the classification of the identified FMDV strains in the current study compared with the other seven FMDV serotypes. The reported sequences in the present study are clustered together with other serotype O FMDV sequences, especially those from Bangladesh.

**Figure 5.**
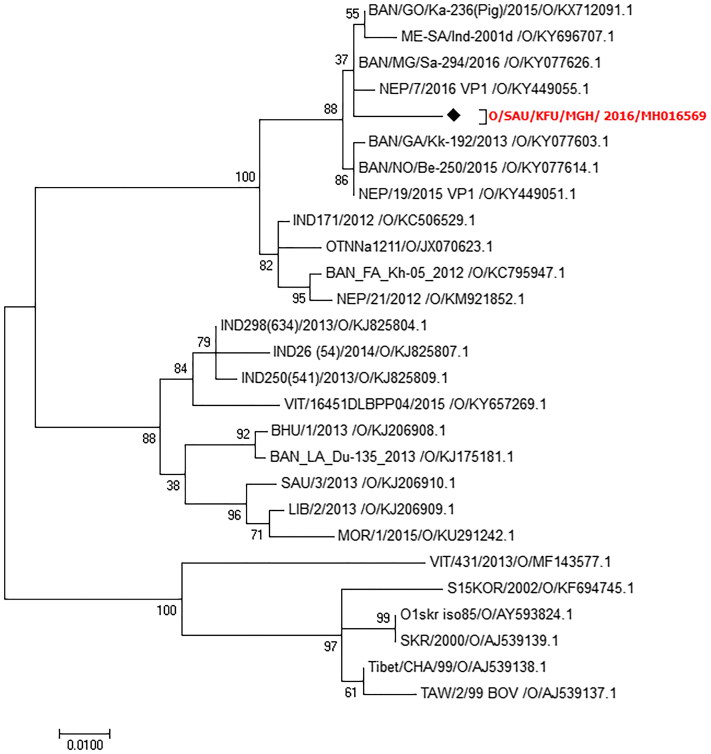
Phylogenetic analysis of the identified FMDV O strains detected in a large ruminant herd in Eastern Saudi Arabia in 2016. The maximum likelihood phylogenetic tree of circulating strains in Eastern Saudi Arabia in 2016 in a dairy herd. The tree was constructed based on the sequences of circulating FMDV serotypes in this herd. The identified serotype shared some variations with the common vaccine strains used in the vaccination of the dairy herd under study, especially the FMDV O strain isolated from cattle in Saudi Arabia in 1993.

We sequenced 13 isolates from the positive FMDV specimens using a partial FMDV VP-1 gene. We assembled and deposited the obtained sequences in GenBank (accession no: MH016569). The phylogenetic analysis based on the partial FMDV VP-1 sequences revealed that the circulating strains in Eastern Saudi Arabia in 2016 belonged to serotype O (Figure 4). These strains showed 99%, 98%, 95%, and 93% nucleotide identity with other FMDV strains from Saudi Arabia isolate-2013 (accession no: MG972596.1), Bangladesh (accession no: KY077626.1), India (accession no: KJ825808.1), and Saudi Arabia (accession no: KJ206910.1), respectively (Figure 5).

### 3.6. Serological surveillance of FMDV

We tested the collected animal sera for the presence of FMDV antibodies and found that 17 animals were positive (22%) (Table 2). In addition, we tested 92 archived cattle serum samples from 1993 and found that 48 of these samples were positive (52%) (Table 2).

## 4. Discussion

FMDV is still one of the most important threats to the bovine industry. Several FMDV strains are currently circulating in many parts in the world, especially in Africa, Asia, and Latin America (Nsamba et al. 2015; Dhikusooka et al. 2016; Brito et al. 2017; Mahapatra et al. 2017; Ali et al. 2018; Hayer et al. 2018; Siddique et al. 2018; Souley Kouato et al. 2018). FMDV infection usually produces lesions in many organs especially the mouths and feet of affected animals. We reported one recent outbreak that occurred late in 2016 in a dairy herd in Saudi Arabia. The affected population showed a high morbidity rate and low mortality. Affected animals showed typical clinical signs of FMDV such as the presence of vesicles on the mouth, tongue, and interdigital spaces. Erosions and ulcerations of the tongue, muzzle, palate, and coronary bands were also reported. These clinical and necropsy findings are very typical and in accord to those previously reported in FMDV infections in cattle (Arzt et al. 2011). We reported the histological progression of FMDV infection in a cattle population during the active course of the viral infection in this particular herd. Lesions started in the form of small papules, which then progressed to vesicles that ruptured and lead to erosions and ulcerations on the mucosa of different organs, especially the lips, muzzle, tongue and coronary bands similar as previously reported in many FMDV outbreaks under both natural and experimental settings (Pacheco et al. 2016; Arzt et al. 2017). Several viral infections are associated with such lesions in the oral cavities of cattle including bovine viral diarrhea/mucosal disease, blue tongue, rinderpest, malignant catarrhal fever, vesicular stomatitis, and FMDV. Unlike other diseases, lesions associated with vesicular stomatitis and FMD usually start as vesicles that subsequently rupture leaving an eroded and ulcerative surface. Our results revealed the presence of intact vesicles or at least the remnants of ruptured ones (Uzal and Hostetter 2016; Gelberg 2017). Both the clinical signs and the histological changes reported in the infected animals were quite similar to other previously described infections with FMDV serotype O in cattle and pigs (Oem et al. 2008). The presence of multifocal lymphocytic and necrotizing myocarditis is a characteristic finding in the hearts of calves and lambs infected with FMDV. Death is mostly attributed to myocarditis, which is usually not accompanied by vesicular lesions (Alexandersen et al. 2003; Gulbahar et al. 2007). To our knowledge, this is the first study to describe the histological changes in FMDV-infected animals in Saudi Arabia in detail. Our serology data showed that 22% of the animals had antibodies against the FMDV 3ABC protein. This indicates that those animals were exposed to a recent natural FMDV infection. Our ELISA testing of the archived cattle sera revealed that 52% of the samples were positive for FMDV 3ABC antibodies. This was in accordance to other FMDV serological surveillances performed in Saudi Arabia in 1988 (Samuel et al. 1988; Bronsvoort et al. 2004; Brito et al. 2017; Mahmoud and Galbat 2017). This suggests that many FMDV strains have circulated in the Saudi Arabia for several decades. This highlights the importance of careful monitoring of FMDV strains by conducting regular molecular and serological surveillance. This will help to monitor the emergence of new strains and fine-tuning the vaccination campaigns across the country. Despite the massive application of FMDV vaccines in endemic regions, several outbreaks have still been reported (Arzt et al. 2011; Stenfeldt et al. 2015; Pacheco et al. 2016). Several FMDV outbreaks have been previously reported in wild and domestic ruminants in the Gulf area and the surrounding countries such as Pakistan, Iraq, Turkey, Iran, and Bangladesh (Klein et al. 2006; Knowles et al. 2009; Baba Sheikh et al. 2017; Mahapatra et al. 2017; Hayer et al. 2018; Siddique et al. 2018). The detection of FMDV in Saudi Arabia began in 1988 when a surveillance study was conducted. Several FMDV outbreaks with different serotypes had been reported previously (Samuel et al. 1988). The FMDV A serotype, which was classified as A/IRN/2005, was reported in Iran during 2005 and then spread to Turkey and Saudi Arabia during 2006 and to Jordan in 2007 (Klein et al. 2007). The full length genome sequence of the FMDV from Saudi Arabia was recently reported in 2015 (Bachanek-Bankowska et al. 2016). The same virus subtype was reported in Bahrain (Knowles et al. 2009). This subtype was modified to generate a new sublineage called A-Iran-05 (ARD-07). It was reported in Turkey and Georgia and became the most predominant subtype in Turkey during 2008 (Knowles et al. 2009). One study reported the presence of a mixed serotype infection in some dairy herds in Saudi Arabia between 1988 and 1991 (Woodbury et al. 1994). This study revealed the circulation of the FMDV serotypes O and Asia 1 among the infected cattle population (Woodbury et al. 1994). The Saudi isolates of the Asia 1 serotype were closely related to Asia 1/Tadzhikistan/64, which was reported in Russia, and Asia 1/TUR/15/73, which was reported in Turkey (Woodbury et al. 1994). Furthermore, the FMDV SAT 2 serotype was detected in Saudi Arabia during 2001 and was closely related to the isolated strains from Eretria (Bronsvoort et al. 2004). The O/ME-SA/Ind-2001 lineage of the virus was reported in both Saudi Arabia and Libya during 2013 (Brito et al. 2017). In the current study, we were able to detect only FMDV serotype O confirmed by sequencing and phylogenetic analysis. The herd under study received the FMDV polyvalent vaccine, which included viruses propagated in the BHK-21 cell line containing A-Iran-05, SAU-95, O-Manisa, O-3039 from Hong Kong, and SAT 2 from Saudi Arabia. Interestingly, reported sequences showed 89% identity to the other two FMDV O serotype candidates present in this vaccine (Manisa and O-3039). Moreover, the reported sequences in this study were quite distinct from the FMDV O serotype previously reported in Saudi Arabia (Valdazo-Gonzalez et al. 2014). The current reported partial VP-1 sequences had a 7% nucleotide difference compared with the last FMDV O strain that circulated in Saudi Arabia in 2013. This confirms the continuous emergence and circulation of FMDV serotype O and its ME-SA/Ind 2001 topotype in Saudi Arabia. We assume this variation among the currently circulating FMDV O strain in Eastern Saudi Arabia and the vaccine strains used may have contributed to this recent outbreak. We believe the preparation FMDV vaccines should be performed with the most recent circulating homologue strains to achieve maximum protection among vaccinated animals. The occurrence of FMDV outbreak in vaccinated animals could be related to many reasons. This animal herd belongs to a group of large dairy farms. They share the common source of ration as well as employee and feed trucks. One possible route of transmission of the virus from one premise to another mechanically is the freely moving the feeding trucks between herds. Veterinarians and other employee could transmit the virus from one group of animals to another mechanically as well. Introduction of new animals during the viral incubation period to a new herd may be another potential source of FMDV transmission. Similar studies reported the mechanical transmission of FMDV mechanically by air, feeding trucks (Paton et al. 2018). The presence of specific antibodies against the FMDV serotype O in dromedary camels in the central region of Saudi Arabia has been described (Yousef et al. 2012). About 6.3% of the tested camel sera were positive for FMDV-O by commercial ELISA (Yousef et al. 2012). Dromedary camels are free moving animals across the desert, which may come in close contact of the FMDV infected cattle or sheep population. This may contributed at least in part to the sustaining and spread of FMDV among certain regions. However, the exact roles of the dromedary camels in the epidemiology of FMDV still need further clarification. We believe the occurrence of FMDV infection in vaccinated animals may be related to the type of the applied vaccine. Presumably, animals were vaccinated with nonhomologous strain of the currently circulating field strains. However, this requires further investigation. Continuous monitoring of the circulating FMDV strains at the molecular level is highly recommended to ensure the selection of the right strain for the preparation of effective vaccines.
